# Body size limits dim-light foraging activity in stingless bees (Apidae: Meliponini)

**DOI:** 10.1007/s00359-016-1118-8

**Published:** 2016-08-05

**Authors:** Martin Streinzer, Werner Huber, Johannes Spaethe

**Affiliations:** 1 Department of Evolutionary Biology, Faculty of Life Sciences, University of Vienna, Althanstraße 14, 1090 Vienna, Austria; 2 Department of Neurobiology, Faculty of Life Sciences, University of Vienna, Althanstraße 14, 1090 Vienna, Austria; 3 Department of Botany and Biodiversity Research, Faculty of Life Sciences, University of Vienna, Rennweg 14, 1030 Vienna, Austria; 4 Behavioral Physiology and Sociobiology, Biozentrum, University of Würzburg, Am Hubland, 97074 Würzburg, Germany

**Keywords:** Meliponini, Light intensity threshold, Vision, Compound eye, Body size

## Abstract

Stingless bees constitute a species-rich tribe of tropical and subtropical eusocial Apidae that act as important pollinators for flowering plants. Many foraging tasks rely on vision, e.g. spatial orientation and detection of food sources and nest entrances. Meliponini workers are usually small, which sets limits on eye morphology and thus quality of vision. Limitations are expected both on acuity, and thus on the ability to detect objects from a distance, as well as on sensitivity, and thus on the foraging time window at dusk and dawn. In this study, we determined light intensity thresholds for flight under dim light conditions in eight stingless bee species in relation to body size in a Neotropical lowland rainforest. Species varied in body size (0.8–1.7 mm thorax-width), and we found a strong negative correlation with light intensity thresholds (0.1–79 lx). Further, we measured eye size, ocelli diameter, ommatidia number, and facet diameter. All parameters significantly correlated with body size. A disproportionately low light intensity threshold in the minute *Trigonisca pipioli*, together with a large eye parameter *P*_eye_ suggests specific adaptations to circumvent the optical constraints imposed by the small body size. We discuss the implications of body size in bees on foraging behavior.

**Electronic supplementary material:**

The online version of this article (doi:10.1007/s00359-016-1118-8) contains supplementary material, which is available to authorized users.

## Introduction

**Fig. 1:**
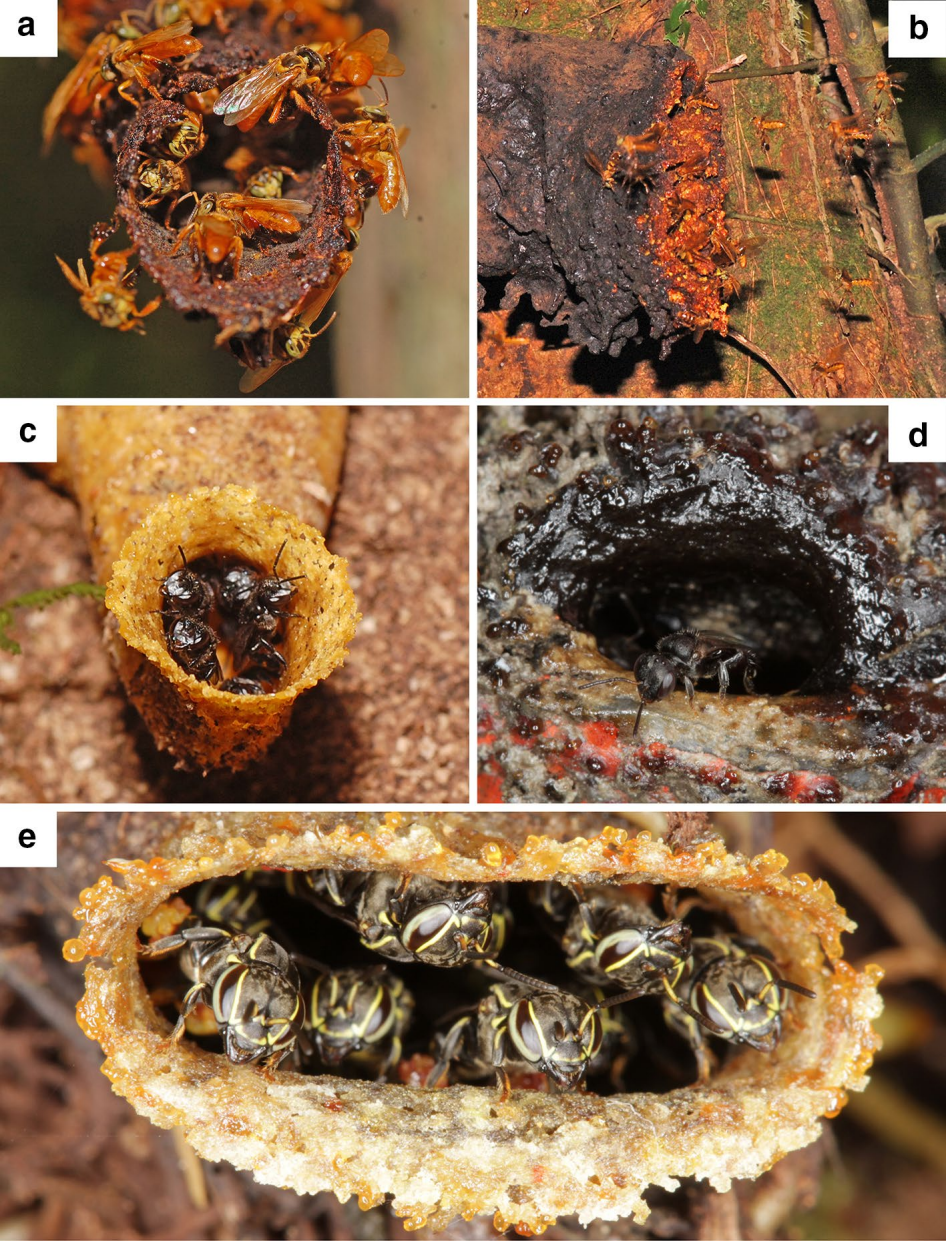
Nest entrances of some of the stingless bee species studied. Nest entrances of **a** *Tetragona ziegleri*, located in a hollow piece of dead wood, **b** *Ptilotrigona occidentalis*, in the trunk of a large living tree, **c** *Scaura argyrea*, in a termite nest, **d** *Trigonisca pipioli*, in a hollow fence post, and **e** *Trigonisca pipioli* in moss surrounding cultivated orchids

Stingless bees (Meliponini) form a monophyletic tribe of the corbiculate bees with a few hundred recognized species in about 60 genera (Rasmussen and Cameron 2010). They inhabit tropical and subtropical regions across the planet. All species are obligatorily eusocial and live in colonies with a few dozen to several thousand members (Wille 1983). As a result of their perennial colony cycle and the large number of foragers in each colony, they represent one of the most abundant pollinator groups of flowering plants in the tropics (Roubik 1989; Heard 1999). Nests are made in cavities in the ground, in tree cavities, abandoned ant or termite nests, or exposed in treetops and crotches (Wille 1983; Roubik 2006; see also Fig. 1). Stingless bees collect nectar and pollen as major food resources that are usually stored in special cells inside the nest. Since the survival, growth and reproductive success of a colony strongly depend on the efficiency of nectar and pollen harvesting, selection is expected to maximize energy influx into the colony by an efficient allocation of the available work force to the available food sources (Michener 1974; Hrncir and Maia-Silva 2013). In several bee species, inter-specific competition is reduced by adjusting the temporal pattern of foraging activity. By shifting the peak activity to dim-light periods, several species manage to harvest ample amounts of nectar and pollen from night flowering plants, thereby escaping competition with other species and evading predators (Wcislo and Tierney 2009). True crepuscular and nocturnal lifestyles evolved repeatedly within bees (Apoidea; Wcislo and Tierney 2009). However, despite the above mentioned adaptive benefits, the majority of bee species is diurnal.

Much of bee behavior outside the nest is partially or entirely guided by vision, such as visual orientation, flight control, detection and recognition of flowers and the nest entrance (Srinivasan 2010). All bees are equipped with apposition compound eyes that consist of several thousand repetitive subunits, called ommatidia (Land 1997). The number of ommatidia determines the number of points sampled in space and thus spatial resolution. Each ommatidium receives light through a small facet lens. The small aperture limits the amount of light that can be collected and thus is a major determinant of light sensitivity. Spatial resolution (number of ommatidia) and light sensitivity (facet diameters) are likely traded-off against each other, depending on the specific lifestyle of the insect (Land 1997). In contrast to superposition compound eyes, the apposition eyes of Apoidea are much less sensitive to light and therefore limit low light activity. This limitation is generally assumed to be the predominant reason for the scarcity of truly nocturnal bees and most species forage only during the bright day hours when enough photons are available for reliable vision (Warrant and Dacke 2011). The few exceptions exhibit a range of specific adaptations that allow the eyes to collect more light by increasing the facet diameter and/or the acceptance angle of the light sensitive rhabdom (Greiner et al. 2004; Somanathan et al. 2009a, b). In addition, neuronal pooling strategies likely further improve the visual abilities (Warrant 1999; Theobald et al. 2006). Larger bee species generally benefit from larger, more acute and more sensitive eyes, which are assumed to be important prerequisites for evolving low-light visual abilities (Land 1997; Kelber et al. 2006). Thus, larger individuals of a species, even without being specifically adapted to low light vision, benefit from a longer foraging period (Kapustjanskij et al. 2007). In contrast to most nocturnal and crepuscular bees, the majority of stingless bee species are small. The largest species, *Melipona* sp., roughly attain the size of honeybee workers, but the majority of species are significantly smaller (Wille 1983; Jarau and Barth 2008).

Body size strongly determines the sensory and physiological capabilities of an individual worker and thus also affects foraging range, flight speed, efficiency by which a certain type of flower can be exploited, and the capability to compete with other species for resources (Wille 1983; Hrncir and Maia-Silva 2013). Stingless bees depend heavily on vision, both for spatial orientation (Hrncir et al. 2003; Eckles et al. 2012), and detection of food sources (Spaethe et al. 2014). However, their small body size (and thus small eye size) likely acts as a constraint on the quality of vision in terms of spatial resolution, light sensitivity and target detection capabilities (Spaethe et al. 2014; Dyer et al. 2016a, b). Consequently, the time window in which foraging is possible may be significantly shortened. Restricted foraging abilities in the morning and evening represent a major disadvantage, since flowers are often rich in pollen and nectar early in the morning before exploitation by flower visitors commences, and late in the evening before night active visitors arrive (Eguiarte et al. 1987; Griebel et al. 1999; Wcislo and Tierney 2009). Eyes with low sensitivity may further impair orientation and nest detection in the dense tropical understory where light levels during the bright day hours are much lower than in open clearings or the canopy region (Endler 1993).

Based on the small body size, we expect that light sensitivity in stingless bees is, on average, lower compared to that of medium to large sized species, such as honeybees and bumblebees, and that within the Meliponini, smaller species are more restricted than larger ones in foraging time. Body size related temporal segregation of foraging has been previously demonstrated (Hrncir and Maia-Silva 2013), but so far it has been attributed mainly to temperature constraints (Pereboom and Biesmeijer 2003), whereas light intensity has rarely been considered as a limiting factor (Velez-Ruiz et al. 2013).

In the present study, we investigated light intensity thresholds for flight activity in several Meliponini species in a tropical lowland rainforest in Costa Rica. The investigated species vary in body size which likely affects their light sensitivity and thus the photic niche that they can utilize. In particular, we asked (1) whether body size determines the photic environment in which the animals are able to forage, (2) how eye size and eye parameters scale with body size in Meliponini, and (3) whether particular adaptations have evolved in the smallest species. To answer these questions, we measured light intensity thresholds for flight activity in eight species of stingless bees and performed detailed morphological investigations on the compound eyes and ocelli of worker bees.

## Methods

### Study site and species

Behavioral observations were performed in the garden or in close vicinity of the tropical field station ‘La Gamba’, Gamba, Puntarenas, Costa Rica (8°42′03″N, 83°12′05″W) in February 2010. The field station borders on the Piedras Blancas National Park and is surrounded by primary and secondary lowland rainforest. Stingless bees are abundant in the vicinity of the station and a number of nests were accessible for observation (Fig. 1). The nest entrances were situated between 0.8 and 3.5 m above ground. In total, 16 nests from eight species were monitored: *Paratrigona opaca* (COCKERELL 1917) (*n* = 6), *Partamona orizabaensis* (STRAND 1919) (*n* = 2), *Ptilotrigona occidentalis* PACKARD 1869 (*n* = 1), *Scaura argyrea* (COCKERELL 1912) (*n* = 2), *Tetragona perangulata* (COCKERELL) 1917 (*n* = 1) *Tetragona ziegleri* (FRIESE 1900) (*n* = 2), *Trigona fulviventris* GUÉRIN-MÉNEVILLE 1845 (*n* = 1) and *Trigonisca pipioli* AYALA 1999 (*n* = 1).

### Light intensity threshold

To determine the onset and offset of flight activity, we monitored nest entrances during one evening and the consecutive morning for each colony. In the evening, observation started between 4:30 and 5:30 p.m. (sunset 5:41 p.m.), depending on the nest location and ambient light level. At that time, bees were active and light levels high enough for them to forage. Species activity (incoming + outgoing bees) was counted in 5 min intervals throughout the observation period. Monitoring proceeded until no more bees entered or left the colony for at least three consecutive 5 min intervals. In the morning, monitoring started at 5:30 a.m. (sunrise 5:51 a.m.). At that time, light levels were below the sensitivity of our meters (see below) and no bees were flying. Monitoring proceeded until stable activity was observed.

Observations took place from a distance using binoculars to leave the colony as undisturbed as possible. For one species, *Ptilotrigona occidentalis*, direct monitoring was impossible due to the aggressive nature of that species and its heavy recruiting behavior (Roubik 2006). Instead, activity was recorded using an infrared-sensitive video camera (Sony DCR-SR65, Tokyo, Japan). Light levels were logged automatically in 30 s intervals. The equipment was set up at least half a day before the evening observation to allow the bees to get used to the set-up. Activity levels were determined from the video sequences, using real time playback and the same counting methods used in the field measurements. For another nest (*Tetragona perangulata)*, video-monitoring was performed due to the inaccessible nest entrance at a height of ~3.5 m above ground.

### Light measurements

Ambient light levels were determined with digital light meters (PCE-174, PCE, Meschede, Germany). The sensitivity threshold of the sensors was 0.1 lx (accuracy ± 0.05 lx). In a few cases (*P. occidentalis*—morning threshold, *T. fulviventris*—morning threshold, *P. orizabaensis*—morning and evening threshold in one of two monitored colonies), bees were able to forage below that threshold. In these cases, we assigned the value 0.1 lx as threshold for the statistical analysis. Light intensities were either recorded manually at the beginning of each 5 min interval or logged automatically in 30 s intervals. The light sensor was directed horizontally, adjacent to and facing away from the nest entrance. Due to the specific arrangement of the lux meter (horizontal rather than vertical), the measured light levels cannot be directly compared to values of vertical illuminance reported in other studies (e.g. Kapustjanskij et al. 2007). Relative values within this study are, however, unaffected, since the same measurement geometry was maintained throughout the observation period.

The presence of the light sensor initially distracted foragers and guards of some species. After a short period, they became accustomed and ignored the sensor. To avoid a bias due to the disturbance, we installed the sensor at least half a day before observations were performed. We assigned the mean of the light intensity value measured for the last interval with species activity and the first without (or vice versa for the morning) as threshold level. Colony thresholds were calculated as the mean of the evening and morning thresholds. In cases, where we monitored more than one colony per species, a species level threshold was calculated as the mean threshold of all colonies of that species.

### Morphometry

Body size differed considerably among Meliponini species and ranged from minute (*T. pipioli,* 2.5–3 mm body length, 0.8 mm thorax width) to medium (*P. occidentalis,* 9–10 mm body length, 1.7 mm thorax width) in our sample (Michener 2007; Jarau and Barth 2008). For detailed morphological measurements, we randomly collected five foragers from one colony of each species. The bees were killed in a freezer and pin-mounted for identification and morphometrics. Measurements were performed on photographs taken with a digital camera (Nikon Coolpix P 5100, Nikon Corporation, Tokyo, Japan) attached to a stereo microscope (Wild Photomacroscope M400, Wild, Heerbrugg, Switzerland). An object micrometer was photographed using identical settings as a reference. All size measurements were performed using ImageJ 1.42 (National Institute of Mental Health, Bethesda, MD, USA). The distance between the wing bases (inter-tegulae span) was used as a reference for body size (Cane 1987). Eye size (length and width) and ocellar diameters were measured as the longest linear distance. Detailed morphological parameters of the compound eye were measured from nail polish replicas of the left eye (Ribi et al. 1989). The replicas were flattened, mounted on microscope slides and photographed in overlapping sections on a light microscope (Zeiss Standard, Carl Zeiss, Oberkochen, Germany), equipped with a digital camera (Basler A312f, Basler, Ahrensburg, Germany). Photographs were stitched using Adobe Photoshop CS4 (Adobe Systems Inc., San Jose, CA, USA). From the eye replicas we determined eye surface area by tracing the outline in ImageJ. Furthermore, we counted all ommatidia and measured the diameter of the largest facets by measuring a row of five ommatidia in all three directions of the hexagonal array and dividing the sum by 15 (Kapustjanskij et al. 2007). In a replica of each studied species, we marked the centers of all ommatidia to create eye maps as visual representation of the distribution of facet diameters. The coordinates of the ommatidia centers were used to calculate the diameter of each individual facet using customized algorithms in ImageJ, MeshLab (Visual Computing Lab–ISRI–CNR, http://meshlab.sourceforge.net/), Python (version 2.7, Python Software Foundation) and CorelDraw X6 (Corel Corporation).

### Statistics

For correlations among and between morphometric parameters and light intensity thresholds, we calculated Pearson’s product moment. All *p* values below 0.05 were considered to be statistically significant. Where multiple comparisons were performed on the same dataset, *p* levels were adjusted using sequential Bonferroni correction. All statistical analyses were performed using R (version 3.1.2; R Development Core Team, 2014).

### Principal component analysis: eye morphology score (EMS)

Since all measured morphological eye parameters significantly correlated with body size (Online Resource 1), we used principal component analysis (PCA) to reduce the dimension of the measured parameters. PCA was performed using the base package in R. Eye surface area was square-root transformed and all morphological eye parameters were normalized prior to the PCA analysis. The first principal component (PC1) was then used as a measure for general eye morphology (eye morphology score, EMS).

### Eye parameter

In addition to the measured morphological parameters, we estimated the eye parameter *P*_eye_ (Snyder 1977). The eye parameter *P*_eye_ describes the relation of facet size and interommatidial angle and is used to describe the trade-off between sensitivity and resolution. Values of ~0.3 indicate that ommatidia operate at the diffraction limit and increasing values imply an increase in light sensitivity. *P*_eye_ differs between species, but also between eye regions, indicating different ecological needs.

*P*_eye_ was estimated as follows:

1 $$P_{\text{eye}} = \Delta \varphi \times d,$$

where Δ*φ* is the average interommatidial angle in radians and *d* the mean facet diameter in micrometers. Since no measurements of Δ*φ* are available for the studied species, the value of Δ*φ* was estimated. As a rough estimate we applied the following equation (Land 1997):

2 $$\Delta \varphi = {\text{sqrt}}\left( {23{,}818/n} \right),$$

where *n* is the number of facets in the compound eye. The equation estimates the global interommatidial angle for a compound eye with a hemispheric visual field. By computing the global interommatidial angle, regional differences in spatial resolution are ignored in our estimate. For consistency, we also used an average facet diameter for the calculation of *P*_eye_. To determine the average facet diameter, we first calculated the average facet area by dividing the eye surface area by the number of ommatidia. From the facet area, we then calculated the facet diameter, i.e. the diameter of the inscribed circle of the hexagonal facet. It must be noted that our estimate of *P*_eye_ only detects species differences at the level of the entire compound eye, and that regional differences within the eye are ignored. To test for significant differences between the studied species’ eye parameters, we performed ANOVA, followed by pairwise *t* tests. *p* levels were adjusted using sequential Bonferroni correction for multiple tests on the same dataset.

### Allometric scaling

To test how eye size scales with body size within Meliponini, we fitted an allometric power function to the raw data (Huxley and Tessier 1936):

3 $$Y = b \times X^{a} ,$$

where *X* is body size (inter-tegulae span), *Y* is eye size (square-root of the eye surface area), *b* is the initial growth index and *a* is the scaling exponent (Huxley and Tessier 1936). We performed linear regression on log_10_ transformed values of inter-tegulae span and the square-root of the eye surface area to calculate the parameters of the allometric power function.

To compare scaling relationships with other bees, we fitted allometric curves to data from Apini and Bombini. These groups were chosen for two reasons. First, together with the Meliponini they represent the three tribes of the eusocial Apidae. The eusocial lifestyle most likely affects selection pressures on the sensory system that probably vary between workers of social and solitary bee species, respectively. Second, for both the Apini (Streinzer et al. 2013) and Bombini (Streinzer and Spaethe 2014), datasets are available that were collected with identical methods.

## Results

### Light intensity thresholds

**Fig. 2:**
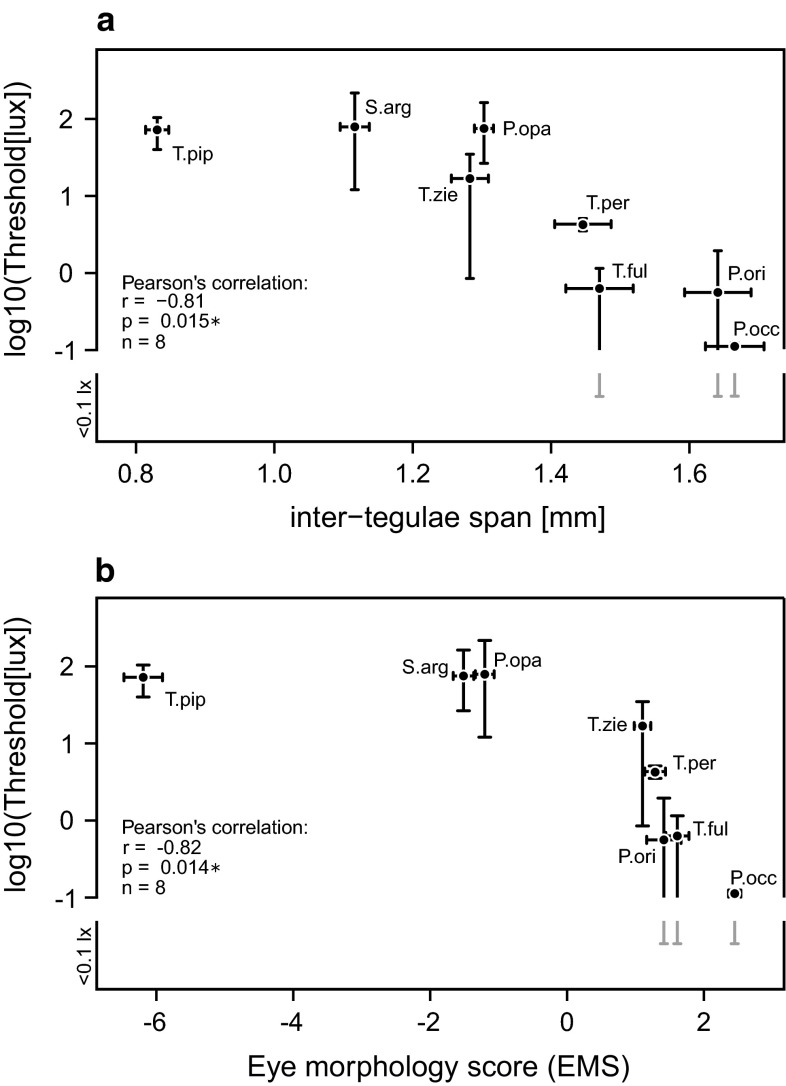
Light intensity threshold plotted as a function of **a** body size and **b** eye morphology. Light thresholds significantly correlated with both parameters. Eye morphology is expressed as the first principal score of a PCA analysis of all measured morphological eye parameters. PC1 explains 96.7 % of the variance in the dataset. Values represent means. The *horizontal error bars* indicate the standard deviation of body size and eye morphology, while the *vertical error bars* represent the total range of observed individual threshold values for each species. Note that the *vertical error bars* are asymmetric due to the logarithmic scaling of the *y*-axis. For three species, we observed flight activity below the sensitivity of our lux meter, which is indicated by a break in the negative *error bar*

As light levels fell in the evening, species activity also decreased until flight activity abruptly stopped. After the cessation of flight activity, some species began to close the nest entrance (e.g. *P. opaca,* pers. obs.). When we started the observation in the morning, usually no or only a few workers were present at the nest entrance. As the light levels increased, more workers appeared at the entrance and in *P. opaca*, workers started to open the nest tube. As soon as the light levels were high enough, workers started to forage and worker flight activity increased steadily. The mean light intensity at which workers started or stopped foraging differed greatly among species, ranging from 0.1 lx in *Ptilotrigona occidentalis* to 79 lx in *Scaura argyrea* (Table 1). No trend was found whether morning (or evening) activity started (or stopped) at lower light intensities (exact binomial test: *p* = 1.0, *n* = 15), and no correlation was found between body size and whether morning or evening thresholds were higher (Pearson’s correlation: *r* = −0.30, *df* = 13, *p* = 0.27). Overall, light intensity thresholds negatively correlated with body size (Pearson’s correlation: *r* = −0.81, *p* < 0.05, *n* = 8; Fig. 2) and eye morphology (EMS, *r* = −0.82, *p* < 0.05, *n* = 8). Eye morphology is expressed as the first principal component (PC1) of all measured morphological parameters, but light intensity thresholds also significantly correlated with any single morphological eye parameter (Online Resource 1).

**Table 1:** Body and morphological eye parameters of the investigated species

Species	Inter-tegulae span (mm)	Eye length (mm)	Eye width (mm)	Eye surface area (mm^2^)	Facet diameter (µm)	Ommatidia (#)	Med. ocellus (mm)	Lat. ocellus (mm)	*P*_eye_ (rad.µm)	Threshold (lx)
*Trigonisca pipioli*	0.83 ± 0.02	0.73 ± 0.01	0.32 ± 0.02	0.24 ± 0.00	13.7 ± 0.6	1524 ± 12	0.10 ± 0.00	0.09 ± 0.00	0.94 ± 0.01	72
	5	5	5	4	4	4	5	5	4	
*Scaura argyrea*	1.12 ± 0.02	1.22 ± 0.03	0.49 ± 0.02	0.77 ± 0.01	17.1 ± 0.2	3171 ± 46	0.17 ± 0.00	0.16 ± 0.01	0.80 ± 0.02	79
	5	5	5	3	3	3	5	5	3	
*Tetragona ziegleri*	1.28 ± 0.02	1.51 ± 0.02	0.67 ± 0.02	1.25 ± 0.02	19.3 ± 0.5	4335 ± 43	0.20 ± 0.01	0.20 ± 0.01	0.75 ± 0.01	17
	5	5	5	5	5	5	5	5	5	
*Paratrigona opaca*	1.30 ± 0.01	1.27 ± 0.02	0.57 ± 0.01	0.72 ± 0.02	16.9 ± 0.5	3014 ± 25	0.15 ± 0.01	0.13 ± 0.00	0.81 ± 0.01	75
	5	5	5	4	4	4	5	5	4	
*Tetragona perangulata*	1.45 ± 0.04	1.61 ± 0.03	0.67 ± 0.03	1.27 ± 0.08	19.4 ± 0.3	4376 ± 62	0.22 ± 0.01	0.20 ± 0.01	0.75 ± 0.02	0.6
	5	5	5	5	5	5	5	5	5	
*Trigona fulviventris*	1.47 ± 0.04	1.69 ± 0.02	0.75 ± 0.02	1.32 ± 0.07	20.4 ± 0.5	4033 ± 61	0.21 ± 0.01	0.21 ± 0.01	0.82 ± 0.01	0.6
	5	5	5	5	5	5	5	5	5	
*Partamona orizabaensis*	1.64 ± 0.04	1.67 ± 0.06	0.68 ± 0.02	1.18 ± 0.06	20.3 ± 0.6	3777 ± 151	0.23 ± 0.01	0.21 ± 0.01	0.83 ± 0.02	1
	5	5	5	5	5	5	5	5	5	
*Ptilotrigona occidentalis*	1.67 ± 0.04	1.82 ± 0.03	0.78 ± 0.01	1.58 ± 0.05	21.5 ± 0.3	4575 ± 66	0.24 ± 0.01	0.22 ± 0.00	0.80 ± 0.02	0.1
	5	5	5	3	3	3	5	5	3	

Values represent mean ± SD. The sample size is given below each value. All morphological eye parameters were measured on the left eye of a specimen

*P*_*eye*_ optical eye parameter

### Body and eye size

Thorax width ranged from c. 0.8 mm in *Trigonisca pipioli* to 1.7 mm in *Ptilotrigona occidentalis* (Table 1). We found significantly positive correlations between any morphological eye parameter and body size (Online Resource 1) with larger species generally possessing larger eyes, more ommatidia, larger facets and larger ocelli. To reduce the number of highly interrelated morphological eye parameters to a single “eye morphology score” (EMS), we performed principal component analysis. PC 1 had similar loadings with identical sign for each of the measured morphological eye parameters and therefore, as body size increases, all the individual parameters also increase at a more or less identical rate. Overall, the EMS parameter explained 96.7 % of the total variance in the dataset (Online Resource 1).

### Eye morphology

**Fig. 3:**
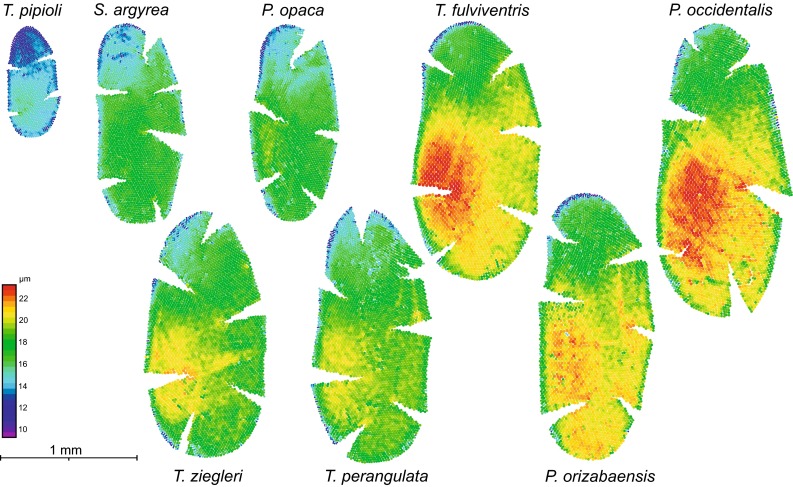
Eye maps of the investigated species. The eye maps illustrate eye size and facet size distribution of all investigated species in ascending order of body size (from *left* to *right*). Each *circle* represents a single ommatidium, where the *size* and *color* indicate facet diameter (false *color scales* on the *left side*). The largest facets are usually found in the fronto-ventral region of the eye. All eye maps are to scale and shown in the same orientation (*left-frontal*, *right-lateral*; *left eye*)

**Fig. 4:**
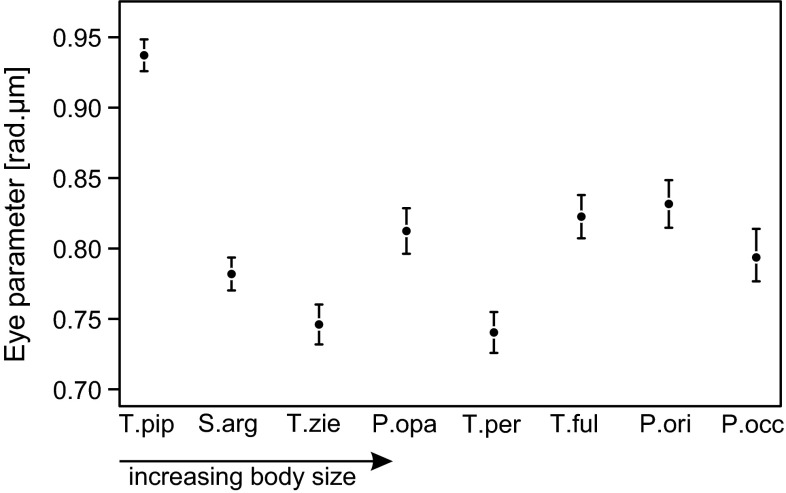
Eye parameter of the investigated species. The eye parameter *P*_eye_ (rad.µm) was calculated from the ommatidia number and mean facet diameter. It represents a measure for facet size in relation to its diffraction limit. A value of 0.3 is the minimum at which the ommatidium operates above its diffraction limit (assuming *λ* = 550 nm; Land 1997). *Higher values* indicate facet diameters that are larger than necessary to avoid diffraction artifacts and hint at a morphological adaptation to increase light sensitivity in very small bees

The compound eyes of all investigated species showed typical eye morphology for bees (Fig. 3). Facet numbers ranged from c. 1500 in *T. pipioli* to c. 4500 in *P. occidentalis* (Table 1). The largest facets are found in the fronto-ventral eye region, while the smallest facets are found in the dorsal region of the compound eye (Fig. 3). The diameter of the largest facets also varied between species and ranged from ~14 µm in *T. pipioli* to ~22 µm in *P. occidentalis* (Table 1). Significant species differences were also found in the calculated eye parameter *P*_eye_. Values were typically in the range of 0.75–0.80 rad.µm, except for *T. pipioli*. For the latter, we calculated an eye parameter of 0.94 ± 0.02 rad.µm (Fig. 4; Table 1). ANOVA followed by pairwise post hoc tests revealed that *P*_eye_ was significantly larger in *T. pipioli* compared to all other species (ANOVA: *F* = 72.9, *df* = 7, *p* < 0.001; pairwise *t* tests between *T. pipioli* and the other species: all *t* > 11.2, all *p* < 0.005 *n* = 7). The large eye parameter nicely correlated with the disproportionately low light intensity threshold found in the smallest species (Fig. 2).

### Allometric scaling

**Fig. 5:**
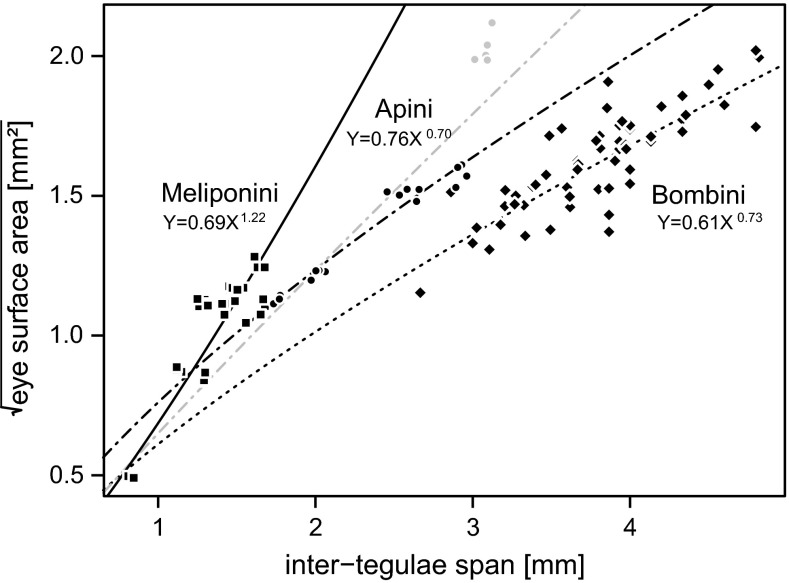
Allometric scaling of eye size in eusocial Apidae. Allometric scaling *curves* were fitted to the dataset of stingless bees (*squares*, *n*_species_ = 8, *n*_individuals_ = 34, *solid line*), honeybees (*circles*, *n*_species_ = 4, *n*_individuals_ = 17, *dash-dot line*) and bumblebees (*diamonds*, *n*_species_ = 11, *n*_individuals_ = 63, *dashed line*). The *grey circles* represent data points of the facultatively crepuscular *A. dorsata* (*n*_individuals_ = 5). The allometric *curve* for Apini including *A. dorsata* is shown in *grey* (*Y* = 0.65.*X* ^0.92^)

Eye size scales allometrically with body size within the investigated Meliponini. Interestingly, the scaling relationship is hyperallometric, i.e. larger species have relatively larger eyes. In contrast, eye size scales hypoallometrically in the two other eusocial Apidae tribes, Apini and Bombini with a scaling exponent of 0.70 and 0.73, respectively (Fig. 5). These tribes differ in the intercept with the *y*-axis, and therefore, honeybees have larger eyes for a given body size than bumblebees. Allometric relationships for *Apis* were calculated only for the strictly diurnal species (*A. andreniformis*, *A. cerana*, *A. florea and A. mellifera*). *Apis dorsata* is facultatively crepuscular and shows distinct adaptations of the eyes (Somanathan et al. 2009b). When *A. dorsata* is included in the analysis, the scaling exponent within Apini is close to one (Fig. 5).

### Influence of the phylogeny

To examine whether there was an influence of the phylogeny of the selected species assembly on the correlation between body size and eye size parameters, we tested the correlations using phylogenetic independent contrasts (Felsenstein 1985). Our results showed identical results to the correlations tested with ordinary statistical methods, suggesting that phylogeny does not bias the interpretation of our results (Online Resource 2).

## Discussion

Stingless bees are a group of predominately small-bodied eusocial insects. Body size limits their environmental activity window, which was previously attributed to ambient temperatures. However, body size also constrains sensory organ morphology and sensory capabilities. In this study, we investigated the hypothesis that the sensitivity of the visual system represents a limiting factor for the foraging behavior in small bodied stingless bee species. We determined light sensitivity thresholds in eight species foraging in a tropical lowland rainforest, where low temperature is not considered a limiting factor. Eye morphology within the studied species positively correlated with body size, suggesting that larger species have more sensitive eyes. The minimum light level necessary for flight differed among species and negatively correlated with body (and thus eye) size. The smallest species in our sample may have evolved specific adaptations, which allow flight at relatively low light levels.

### Body size

Stingless bees show a large variation in body size; species range from about the size of *Drosophila* to small honeybee workers (Jarau and Barth 2008). Compared with the other tribes of eusocial bees, Meliponini are on average smaller. As eusocial animals, the success of the colony critically depends on the amount of nutrients that workers are able to gather (Michener 1974). Several size-related factors have been identified previously, which influence the foraging performance of a colony. Larger species have longer flight ranges and can thus cover a larger area while searching for profitable food sources (Roubik and Aluja 1983; Wille 1983; Araújo et al. 2004). Furthermore, larger bees are able to collect greater amounts of nutrients per unit time (Spaethe and Weidenmüller 2002). Another benefit of having larger bodies is the ability to forage at lower ambient temperatures (Heinrich 1995; Teixeira and Campos 2005) and thus earlier in the morning or during periods of unfavorable weather conditions. Nonetheless, large bodies may constrain foraging during the day when ambient temperatures are too high (Pereboom and Biesmeijer 2003).

Body size is further known to correlate with sensory organ size and sensitivity, and larger species/individuals often have improved sensory capabilities (Jander and Jander 2002; Spaethe and Chittka 2003; Kapustjanskij et al. 2007; Spaethe et al. 2007). Higher resolution and sensitivity of their sensory organs thus may allow them to detect food sources from a larger distance and with higher accuracy (Spaethe and Chittka 2003; Dyer et al. 2008). In spite of these advantages, there must be benefits of having a small body size, since it evolved several times independently within Meliponini (Online Resource 2; Pignata and Diniz-Filho 1996; Michener 2001). Such benefits likely include better availability of suitable nesting sites (Inoue et al. 1993; Michener 2001) and improved survival during periods with limited resources (Quezada-Euán et al. 2010). However, miniaturization may have been possible only through reduced selection on thermoregulatory factors, which restricts the distribution of Meliponini to lowland regions in tropical and subtropical habitats (Heinrich 1995; Pereboom and Biesmeijer 2003).

### Flight threshold

Body size related temporal segregation of foraging has been demonstrated in stingless bees and has been attributed to the ability to fly at different ambient temperatures (Teixeira and Campos 2005; Hrncir and Maia-Silva 2013). In our study, we also found size-dependent differences in the timing of foraging onset and cessation. During the study period temperatures never fell below 24 °C (mean minimum daily temperature 25 °C), which is above the lower temperature limit for flight measured in a variety of stingless bees of different body sizes (Teixeira and Campos 2005; Norgate et al. 2010; Maia-Silva et al. 2014). All bee species were highly mobile before foraging began in the morning, e.g. they bustled around the nest entrance, but initiated flight only when the light levels reached a critical value. If morning temperatures limited flight initiation, we would expect higher light intensity thresholds in the morning, compared to the evening when temperatures were on average 10 °C higher. However, we found no time or body size related differences of light intensity threshold. Therefore, we assume that light intensity is the major determinant of flight onset and stoppage for a colony, similar to the tropical nocturnal bee *Megalopta genalis* (Kelber et al. 2006).

### Eye morphology and light sensitivity

In our study, we found evidence that eye morphology determines the environmental window in which stingless bee workers are able to forage. The minimum light intensity necessary for flight negatively correlated with body size and eye morphology, respectively (Fig. 2). While the largest species are already active at light levels similar to a full moon under clear sky (Johnsen et al. 2006), the smallest species (*P. opaca, S. argyrea, T. pipioli*) need light intensities a few orders of magnitude higher (Fig. 2; Table 1). Striking is that the threshold of the smallest investigated species, *T. pipioli*, is not higher than that of two larger species, *S. argyrea* and *P. opaca*, despite its smaller eyes, ocelli and facet lenses (Figs. 2, 3; Online Resource 1). Due to the quadratic relationship between facet diameter and light catch, each ommatidium of *T. pipioli* captures only 40 % of the photons compared to ommatidia of *P. occidentalis*. The reduced light sensitivity might be counteracted by increasing acceptance angles. Acceptance angles and inter-ommatidial angles are usually closely linked in diurnal bees, but are greatly enlarged in night active bees and wasps to increase photon catch (Greiner et al. 2004; Warrant et al. 2004; Greiner 2006; Somanathan et al. 2009a, b). To estimate overall light sensitivity of a compound eye, we additionally calculated the eye parameter *P*_eye_ for all species and found a significantly larger value for *T. pipioli* than for all other bees (Fig. 4). Values above 0.9 are only observed in crepuscular or nocturnal bees (Jander and Jander 2002), which indicates that *T. pipioli* has eyes adapted for light sensitivity at the expense of spatial resolution to compensate for limitations imposed by small eyes. This result highlights the importance of sensitive eyes when foraging in dense rainforest habitats. In addition to morphological adaptations, it is also possible that secondary neural strategies, such as spatial and temporal pooling of receptor signals, further improve the information sampled by the compound eye (Warrant et al. 1996; Warrant 1999; Greiner et al. 2005; Theobald et al. 2006).

The flight capabilities of bees are further influenced by information gathered by the three simple lens eyes on the vertex, the ocelli. They are assumed to play a role in flight stabilization and orientation (Wellington 1974; Wilson 1978; Mizunami 1995). Wellington (1974) showed that flight and straight-line orientation can be accomplished using only the ocelli, but spatial vision of the compound eye is necessary for landmark orientation, height estimation and nest detection. It is likely that the two visual systems interact and that the more sensitive ocelli allow the insects to traverse small flight distances in regions that are too shady for reliable spatial vision. The importance of large ocelli is highlighted by their huge size and specific adaptations in nocturnal hymenopterans (Kerfoot 1967; Greiner et al. 2004; Greiner 2006; Warrant et al. 2006; Berry et al. 2011). However, in our species sample, ocelli are not particularly enlarged (Online Resource 1; Table 1), and the ratio of ocelli to body size appears similar to that in other stingless bees (Ribi et al. 1989).

### Ecology and behavior

Our results show that flight activity in small stingless bees is restricted by light levels, and thus they are only able to start foraging later in the morning and stop earlier in the evening than large bees. In bumblebees, it has been shown that larger individuals with more sensitive eyes are able to forage for about 10–15 min longer in the morning and evening (Kapustjanskij et al. 2007). Based on our observations, we roughly estimated that the larger bee species are able to forage between 30 min and 1 h longer in the morning and evening. This most likely confers benefits to the larger species since several flowers accumulate nectar and pollen during the night, which can be exploited by the first species that arrive in the morning (Griebel et al. 1999). Likewise, resources from flowers that open in the evening can only be collected by species that capable of flight at lower light intensities (Eguiarte et al. 1987), see also discussion in (Kelber et al. 2006; Wcislo and Tierney 2009). Kelber et al. (2006) argued that the most critical phase of foraging, in terms of light levels, is orientation at the nest entrance, since light levels are well below those observed in the canopy where the bees forage (Endler 1993). It is possible that the low visual sensitivity of the small species limits their ability to select nesting sites in the darkest regions of the tropical rainforest, where lighting conditions would restrict the activity period to a few very bright hours of the day. However, whether the photic environment plays a role in nest site choice is a question that must be answered in future studies.

### Eye scaling within eusocial Apidae

Jander and Jander (2002) investigated the relationship between body size and eye morphology in 15 taxa of mostly solitary bees. Their results showed that eye size scales hypoallometrically (scaling exponent <1.0) with body size, i.e. larger individuals have relatively smaller eyes. In our sample, stingless bee eye size scaled with an exponent of 1.22, thus larger species have relatively larger eyes. Differences in scaling are expected between solitary and social species, since sterile workers likely can relax selection on fecundity and allocate more resources to sensory processing (del Castillo and Fairbairn 2011; Streinzer et al. 2013; Streinzer and Spaethe 2014). To investigate whether different scaling rules apply in eusocial bee workers, we compared the results from stingless bees to two datasets of the other two tribes of eusocial Apidae, honeybees (Streinzer et al. 2013) and bumblebees (Streinzer and Spaethe 2014). Interestingly, in both groups eye size scales hypoallometrically with body size, similar to the results of Jander and Jander (2002). In stingless bees we found hyperallometric scaling of eye size (scaling exponent >1.0). This result is surprising, since our experiments suggest that smaller species should invest disproportionately in their visual organs to counteract the low sensitivity of their eyes.

One possible explanation is that the contribution of sensory and brain structures which cannot be arbitrarily scaled (e.g. Mares et al. 2005) set an upper limit to the space that can be attributed to modular units, such as the visual and olfactory sense organs (Chittka and Niven 2009, but see O’Donnell et al. 2011). It is interesting to note that Seid et al. (2011) reported that extremely small individuals and species in ants have relatively smaller brains than expected if scaling followed the allometric slope of the larger species (see also discussion in Eberhard and Wcislo 2011). Future studies including a larger sample size comprising both larger Meliponini species and smaller Bombini individuals will have to determine, whether the observed grade changes are related to phylogeny or body size per se (Eberhard and Wcislo 2011).

## Conclusion

Stingless bees are a group of comparatively small eusocial bee species. Here, we showed that eye morphology, which is constrained by body size, significantly influences the light levels at which the bees are able to fly. Large species with higher light sensitivity benefit from an extended period of foraging and can thus exploit food sources that are rich in nectar and pollen both in the early morning and late evening. We hypothesize that, at least, in tropical lowland environments where temperatures do not restrict flight initiation of the smaller species, light intensity is the major factor that leads to temporal segregation of the onset of foraging. Data on the minute *T. pipioli* also suggest that the smallest species might have evolved specific adaptations that increase light sensitivity to cope with the challenges of flight in the dark rainforest, even under daylight conditions. Our results further highlight the need for future studies to better understand how small diurnal (stingless) bees cope with challenges of vision.

## Electronic supplementary material

Below is the link to the electronic supplementary material.

Supplementary material 1 (PDF 401 kb)

Supplementary material 2 (PDF 182 kb)
