# Supplementary material for: Body size limits dim-light foraging activity in stingless bees (Apidae: Meliponini)
Source: J Comp Physiol A Neuroethol Sens Neural Behav Physiol. 2016 Aug 5;202(9):643–55. doi: 10.1007/s00359-016-1118-8 (PMC5023731; doi:10.1007/s00359-016-1118-8)
Supplement: Supplementary file 1 — Supplementary material 1 (PDF 401 kb) [file 359_2016_1118_MOESM1_ESM.pdf]

Body size limits dim-light foraging activity in stingless bees (Apidae: Meliponini)
Journal of Comparative Physiology A
Martin Streinzer*, Werner Huber and Johannes Spaethe

*correspondence:
Dr. Martin Streinzer
Department of Neurobiology, Faculty of Life Sciences, University of Vienna
email: martin.streinzer@univie.ac.at

Online Resource 1

Tab. S1 Factor loadings of the principal component analysis (PCA)

parameter	PC loading
eye surface area	0.382
eye length	0.382
eye width	0.376
median ocellus	0.376
lateral ocellus	0.378
facet diameter	0.376
# ommatidia	0.376

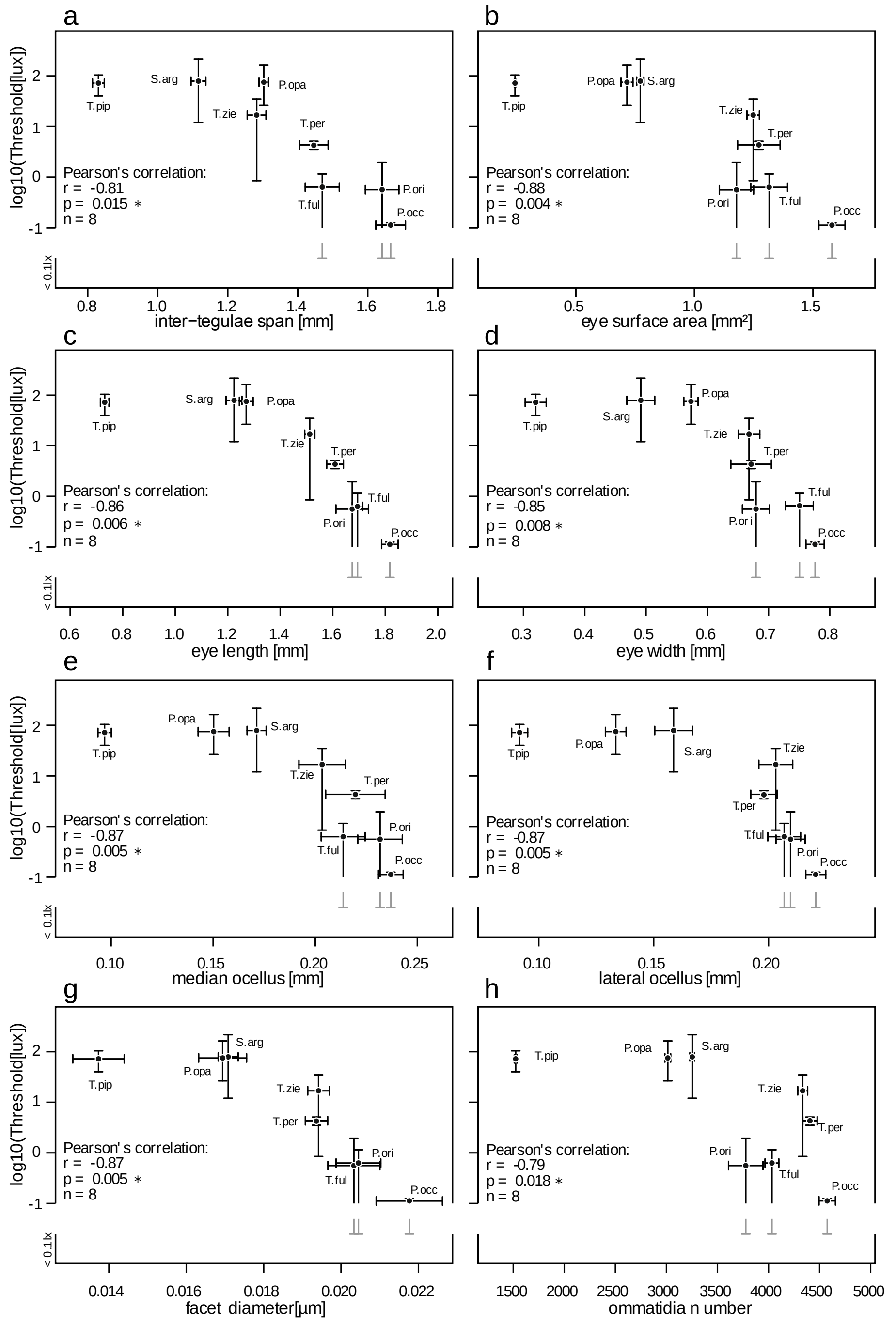

Fig. S1 Correlation of eye parameters with light intensity thresholds

Light intensity threshold plotted against (a) body size (inter-tegulae span), (b) eye surface area, (c) eye length, (d) eye width, (e) median ocellus diameter, (g) lateral ocellus diameter, (h) facet diameter and (i) number of ommatidia. For sample sizes, see Table 1. All eye parameters correlate significantly with the light intensity threshold. Uncorrected *p*-values are given in the graphs; an asterisk indicates significant correlation after sequential Bonferroni correction. Values represent means. The horizontal error bars indicate the standard deviation of body size and eye morphology parameters, while the vertical error bars represent the total range of observed individual threshold values for each species. Note that the vertical error bars are asymmetric due to the logarithmic scaling of the y-axis. For three species, we observed flight activity below the sensitivity of our lux meter, which is indicated by a break in the negative error bar. T.pip – *Trigonisca pipioli*, S.arg – *Scaura argyrea*, T.zig – *Tetragona ziegleri*, P.opa – *Paratrigona opaca*, T.per – *Tetragona perangulata*, T.ful – *Trigona fulviventris*, P.ori – *Partamona orizabaensis*, P.occ – *Ptilotrigona occidentalis*.

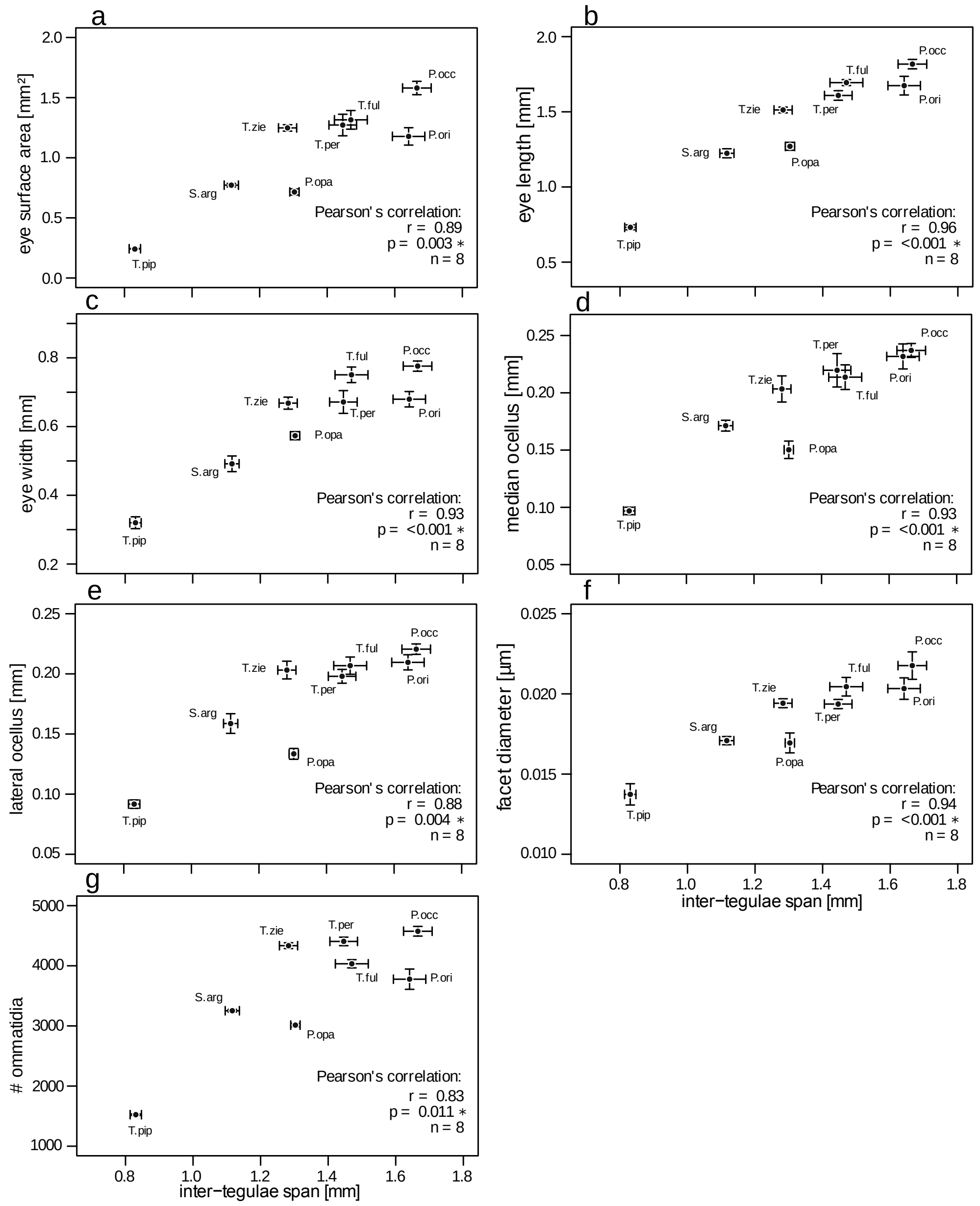

Fig. S2 Correlation of eye parameters with body size

Inter-tegulae span plotted against (a) eye surface area, (b) eye length, (c) eye width, (d) median ocellus diameter, (e) lateral ocellus diameter, (f) facet diameter and (g) number of ommatidia. For sample sizes, see Table 1. All eye measures correlate significantly with inter-tegulae span. Uncorrected *p*-values are given in the graphs; an asterisk indicates significant correlation after sequential Bonferroni correction. Values represent means. The error bars indicate the standard deviation of the measured morphological parameter. T.pip – *Trigonisca pipioli*, S.arg – *Scaura argyrea*, T.zig – *Tetragona ziegleri*, P.opa – *Paratrigona opaca*, T.per – *Tetragona perangulata*, T.ful – *Trigona fulviventris*, P.ori – *Partamona orizabaensis*, P.occ – *Ptilotrigona occidentalis*.
