# Supplementary material for: Body size limits dim-light foraging activity in stingless bees (Apidae: Meliponini)
Source: J Comp Physiol A Neuroethol Sens Neural Behav Physiol. 2016 Aug 5;202(9):643–55. doi: 10.1007/s00359-016-1118-8 (PMC5023731; doi:10.1007/s00359-016-1118-8)
Supplement: Supplementary file 2 — Supplementary material 2 (PDF 182 kb) [file 359_2016_1118_MOESM2_ESM.pdf]

Body size limits dim-light foraging activity in stingless bees (Apidae: Meliponini)
Journal of Comparative Physiology A
Martin Streinzer*, Werner Huber and Johannes Spaethe

*correspondence:
Dr. Martin Streinzer
Department of Neurobiology, Faculty of Life Sciences, University of Vienna
email: martin.streinzer@univie.ac.at

Online Resource 2

Material & Methods

Phylogenetic independent contrasts (PIC)

To account for the phylogenetic background of the investigated species, phylogeny informed statistics were applied, in addition to standard procedures (Felsenstein 1985). The phylogenetic tree used in the analysis was pruned from the time-calibrated phylogeny published by Rasmussen *et al.* (Rasmussen and Cameron 2010). For cases where the study species was not included in the original phylogeny, congeners were used instead. Statistical analyses were performed on phylogenetically independent contrasts (PIC) of $\log_{10}$ transformed original data. Eye surface area was square-root transformed prior to the analysis. PICs were calculated in R (version 3.1.2; R Development Core Team, 2014) using the APE package (Paradis et al. 2004). Correlation between the contrasts of body size and the tested eye parameters were calculated using the R package PHYLOGR (version 1.0.8). All correlations were forced through the origin (Garland et al. 2005). *p*-values below 0.05 were considered statistically significant. For multiple comparisons, *p*-values were adjusted using sequential Bonferroni correction.

Ancestral state reconstruction

To better understand how worker body size has evolved within the Meliponini, we modelled the ancestral states of the body sizes of our study species. Ancestral states were reconstructed as continuous character from the species mean of the inter-tegulae span. We calculated root-node characters using phylogenetic independent contrasts (Felsenstein 1985) using the APE package in R.

Results

PIC

None of the tested eye parameters yielded a significant correlation between the absolute contrasts and their standard deviation. Therefore, one of the basic assumptions of phylogenetic independent contrasts is met (Felsenstein 1985; Garland et al. 2005).

All measured eye parameter correlated significantly with body size contrasts (Tab. S2), supporting the interpretation that evolutionary change of body size is accompanied by a change in all eye parameters.

Ancestral state reconstruction

The reconstructed node values for body size show frequent changes of body size in both directions during species evolution (Fig. S3). According to the reconstruction, the smallest species in our sample, *T. pipioli*, evolved from a larger ancestor.

References

Felsenstein J (1985) Phylogenies and the comparative method Am Nat 125:1-15 doi:10.2307/2461605
Garland T, Jr., Bennett AF, Rezende EL (2005) Phylogenetic approaches in comparative physiology J Exp Biol 208:3015-3035 doi:10.1242/jeb.01745
Paradis E, Claude J, Strimmer K (2004) APE: Analyses of phylogenetics and evolution in R language Bioinformatics 20:289-290 doi:10.1093/bioinformatics/btg412
Rasmussen C, Cameron SA (2010) Global stingless bee phylogeny supports ancient divergence, vicariance, and long distance dispersal Biol J Linn Soc 99:206-232 doi:10.1111/j.1095-8312.2009.01341.x

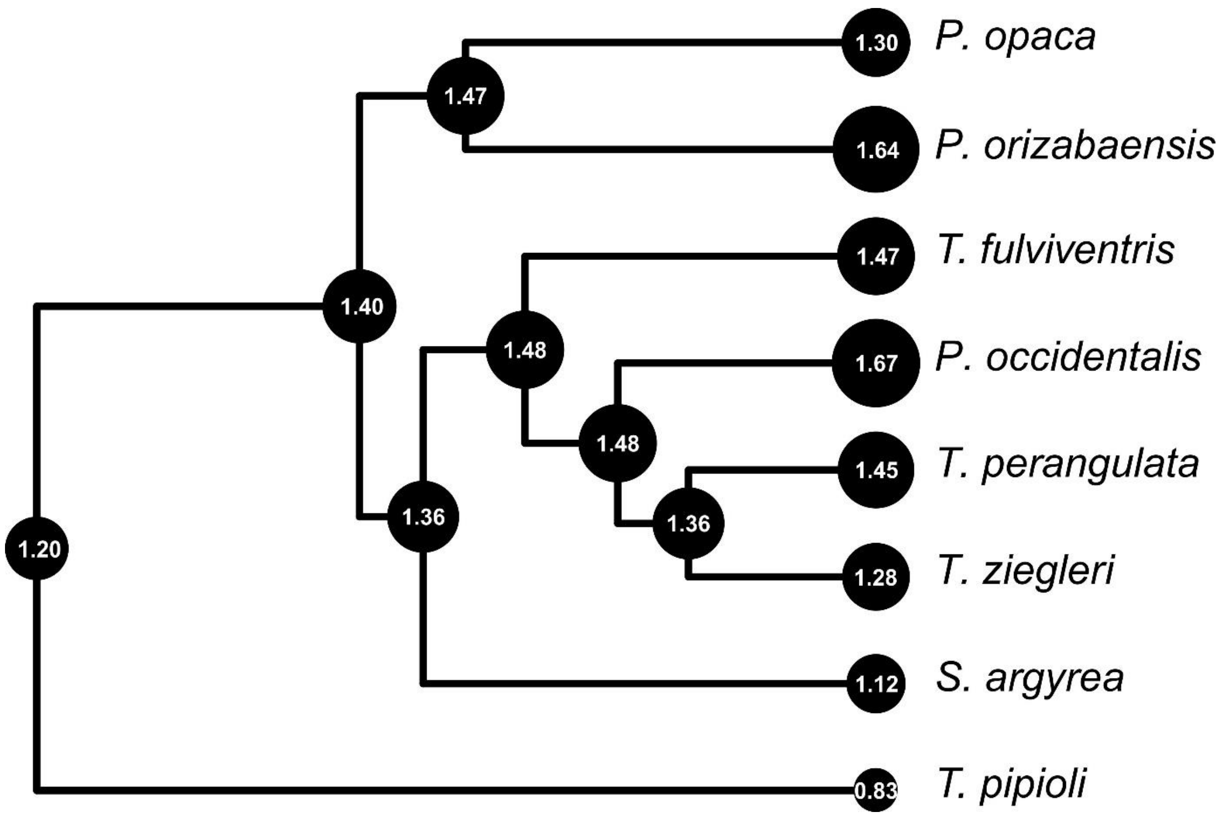

Fig. S3 Phylogeny of the studied species
The phylogeny was used for the calculation of PICs. Modeled ancestral character states for inter-tegulae span are indicated for all tips and root nodes. Scaled circles are used as visual representation of modeled body sizes.

Tab. S2 Correlation between phylogenetic independent contrasts of body size *vs.* eye parameters

parameter	ρ	*P*
eye surface area	0.80	<0.0001*
eye length	0.90	<0.0001*
eye width	0.85	<0.0001*
median ocellus	0.85	<0.0001*
lateral ocellus	0.75	<0.0001*
facet diameter	0.88	<0.0001*
# ommatidia	0.72	<0.001*

Correlation between body size contrasts and eye value contrasts were forced through the origin. Unadjusted *p*-values are presented. *p*-values that are significant after sequential Bonferroni correction are marked with an asterisk.
